# Development of a Novel Lipophilic, Magnetic Nanoparticle for *in Vivo* Drug Delivery

**DOI:** 10.3390/pharmaceutics5020246

**Published:** 2013-04-23

**Authors:** Thomas Linemann, Louiza B. Thomsen, Kristian G. Du Jardin, Jens C. Laursen, Jesper B. Jensen, Jacek Lichota, Torben Moos

**Affiliations:** Section of Neurobiology, Biomedicine, Department of Health Science and Technology, Aalborg University, Fr. Bajers Vej 3B, 1.216, DK-9220 Aalborg East, Denmark; E-Mails: thomas@linemann.com (T.L.); lbt@hst.aau.dk (L.B.T.); du_jardin@mail.com (C.G.J.); jcl@hst.aau.dk (J.C.L.); moos@os.dk (J.B.J.); jlichota@hst.aau.dk (J.L.)

**Keywords:** blood-brain barrier, endothelium, magnetofection, magnetic field, nanoparticle, transfection

## Abstract

The aim of the present study was to evaluate the transfection potential of chitosan-coated, green-fluorescent magnetic nanoparticles (MNPs) (chi-MNPs) after encapsulation inside polyethylglycol (PEG)ylated liposomes that produced lipid-encapsulated chitosan-coated MNPs (lip-MNPs), and also to evaluate how these particles would distribute *in vivo* after systemic injection. The transfection potential of both chi-MNPs and lip-MNPs was evaluated *in vitro* in rat brain endothelial 4 (RBE4) cells with and without applying a magnetic field. Subsequently, the MNPs were evaluated *in vivo* in young rats. The *in vitro* investigations revealed that the application of a magnetic field resulted in an increased cellular uptake of the particles. The lip-MNPs were able to transfect the RBE4 cells with an incidence of approximately 20% of a commercial transfection agent. The *in vivo* distribution studies revealed that lip-MNPs had superior pharmacokinetic properties due to evasion of the RES, including hepatic Kuppfer cells and macrophages in the spleen. In conclusion, we were able to design a novel lipid-encapsulated MNP with the ability to carry genetic material, with favorable pharmacokinetic properties, and under the influence of a magnetic field with the capability to mediate transfection *in vitro.*

## 1. Introduction

The usage of magnetic nanoparticles (MNPs) for drug delivery receives much attention because they bring significant advantages for targeted delivery with increases in drug concentration at target sites and reduced drug deposition off-target [1,2]. They have been used for delivery of a plethora of drugs and especially delivery of anti-cancer drugs dominates [3,4]. A relatively new application of the MNPs is their use as a carrier for non-viral vectors intended for gene delivery and therapy, a process commonly referred to as magnetofection [5]. The concept of using MNPs for such purpose is generally identical to that of their application in targeted drug delivery, where an external or implanted magnet is used to attract the MNPs to the target site, which allows for targeted delivery of genetic material such as plasmid DNA.

MNPs are comprised of a centrally located magnetic core, which may be surrounded by a variety of protective and/or functionally active molecules. The magnetic core may be composed of various magnetic materials, however, the magnetic iron-oxide core is the most commonly used since it seems to be the least toxic [1,2]. This magnetic property can assist in targeting the MNPs to the site of interest and has been applied both in vitro and in vivo for several biomedical applications. The surface coating of the MNPs also attains a pivotal role in experimental assays, as it is important not only for their functionality but also for prevention of unwanted effects such as agglomeration, interaction with serum proteins or capture by the reticuloendothelial system (RES) leading to rapid clearing following systemic administration [3,4,5]. Hence, surface coating of the MNPs is crucial for both efficacy, but also safety, and therefore surface modification with biocompatible coatings are extensively studied. Manipulation of the surface of MNPs also allows for tailored design of the MNPs for specific demands, e.g., design of nanocontainers and delivery systems for gene therapy where sufficient amounts of cationic particles are needed for DNA complexation and condensation to achieve successful transfection.

Liposomes are non-toxic, biocompatible, and biodegradable vesicles, which surround and encapsulate an inner aqueous compartment. Traditionally, liposomes composed of phospholipids and cholesterol, suffer from a high plasma clearance due to rapid removal by the RES, which is a significant drawback for utilization *in vivo* [6,7]. Since the percentage of liposomes delivered to a target organ is directly dependent on the available concentration in plasma, it is desirable to increase plasma half-live which can be achieved by incorporation of polyethylene glycol (PEG) derivatized lipids within the phospholipid bilayer. Cationic liposomes have been used as non-viral transfection vector for several years. The mechanism, by which cationic liposomes facilitate transfection, generally resides on their ability to associate with the negative cell membrane by electrostatic interactions, which is proceeded by endocytosis of the liposome. Subsequently, the cationic lipids destabilize the resulting endosomal membrane and the genetic material is released into the cytosol of the cells [6,7]. The entry into the nucleus is thought to take place either through passive entry during cell division or through active transport through nuclear pores. After entering the cell nucleus, the genetic material may be transcribed followed by translation into a therapeutic protein, and both processes are mediated by the host transcription and translation machineries, respectively.

The aim of the present study was to evaluate the transfection potential of various MNPs based formulations encapsulating the MNPs inside liposomes and also to evaluate how these particles would distribute *in vivo* after systemic injection. The magnetofection potential was investigated using plasmid DNA HcRed-C1, which encodes the far-red fluorescent protein HcRed [8]. The drug carriers used for gene delivery in the transfection studies were commercially available chitosan-coated MNPs (chi-MNPs) and lipid-encapsulated chitosan-coated MNPs (lip-MNPs). The use of chi-MNPs was based on the findings from Kievit *et al*. [9], who concluded that this specific surface coating offers an improved safety profile and prevents unwanted aggregation, thus potentiating the overall efficacy.

## 2. Materials and Methods

### 2.1. Preparation of Lipid-Encapsulated Magnetic Nanoparticles

**Figure 1:**
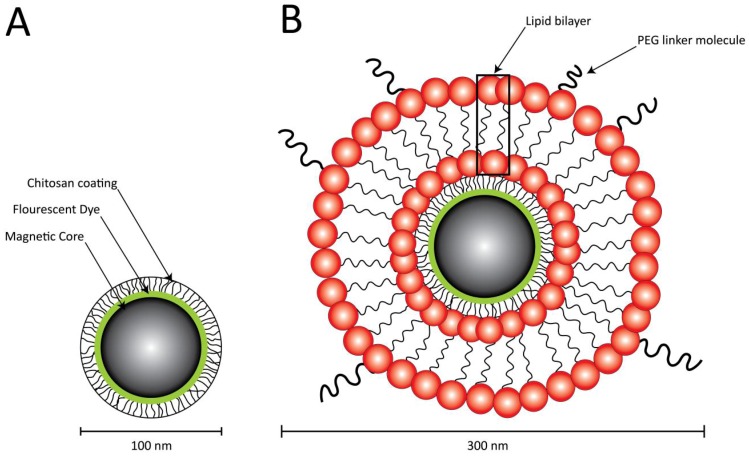
Principal structure of the two magnetic nanoparticles (MNPs) used in the present study. (**A**) The chi-MNP consists of a magnetic iron-oxide core covered by a lipophilic green fluorescent dye and a second layer of chitosan coat that prevents aggregation with other MNPs; (**B**) The lip-MNP is made from the chi-MNP by encapsulation inside a liposome. The liposomes were additionally PEGylated for use *in vivo*.

Chitosan-coated, green-fluorescent MNPs (chi-MNPs) (Chemicell, Cat. No 4418-1) were lipid-encapsulated to form lipidized-MNPs (lip-MNPs) (Figure 1) based on the method presented by Pan *et al*. [10]. A mixture consisting of l-α-phosphatidylcholine (Soy PC) (Avanti, Cat. No 840072), dimethyldioctadecylammonium bromide (DDAB) (Sigma, Cat. No D2779-10G, Brondby, Denmark), and 1,2-distearoyl-*sn*-glycero-3-phosphoethanolamine-*N*-[maleimide (polyethylene glycol)-2000] (DSPE-PEG2000-MAL) (Avanti, Cat. No 880126, Instruchemie BV, Delfzyl, The Netherlands), all dissolved in chloroform, was prepared in a mole ratio of 37:60:3, respectively. This lipid suspension was dried in a round-bottom bottle to form a thin lipid film under continuous gaseous N_2_ supply. To ensure complete evaporation of the organic solvent, the resulting lipid film was additionally exposed to gaseous N_2_ for 30 min. The resulting lipid film was rehydrated in HEPES buffer consisting of 136 mM NaCl, 10 mM HEPES, and 1 mM EDTA containing the MNPs in a *w*/*w* ratio of 1:10 (MNPs/solid lipid). To facilitate complete dissolution of the lipid film, the mixture was immediately vortexed for 5 min, after where the mixture was placed on a rocking plate, and incubated at ambient temperature for a maximum of 2 h to allow complete rehydration. Next, the mixture was placed in a water bath and extensively sonicated (Bransonic, 1510E-DTH) for 2 h to break up any lipid aggregates, and to promote the formation of unilamellar liposomes. Excess lipid was removed by magnetic decantation and the resulting lip-MNPs characterized by means of size and ζ-potential. The particles were stored at 4 °C for a maximum of 3 days before utilization.

### 2.2. Size and the ζ-Potential Particle Characterization

The size and charge of the chi-MNPs and lip-MNPs were assessed using Zetasizer Nano ZS (Malvern, Malvern Instruments Nordic AB, Greve, Denmark) in where the hydrodynamic diameter of the particles is measured by means of dynamic light scattering (DLS), and their surface charge measured by Laser Doppler electrophoresis and expressed as the ζ-potential. The resulting data were analyzed using Malvern Zetasizer Software v. 6.2 (Malvern Instruments Nordic AB, Greve, Denmark). All measurements were performed on three separately prepared samples.

### 2.3. Assessment of DNA Binding Capacity

The DNA binding capacity of chi-MNPs and lip-MNPs was assessed in a series of gel retardation assays. Different *w*/*w* ratios of pDNA/MNP were incubated at room temperature for 20 min to allow complexation by adsorption and subsequently loaded in separate wells on a 1% agarose gel. A tris-acetate-edta (TAE) solution was used as running buffer and electrophoresis performed for 30 min at 50 V. pDNA bands were visualized with ethidium bromide (0.5 µg/mL). The gel was imaged with Kodak Image Station 4000MM Pro. (Carestream Health, Skovlunde, Denmark), and data analyzed using appropriate software (Kodak Molecular Imaging Software, v. 5.0.0.86, Carestream Health, Skovlunde, Denmark). The highest pDNA/MNP ratio for each vector was used in the subsequent transfection studies.

### 2.4. *In Vitro* Transfection Studies

#### 2.4.1. Cell Culture

Immortalized RBE4 cells were cultured at 37 °C, 5% CO_2_ in a humidified atmosphere (Holm and Halby, IGO 150 cell life, Brondby, Denmark) using a growth medium consisting of 50% Alpha-MEM with Glutamax-1 (Gibco, Cat. No 32-561, Life Technologies, Naerum, Denmark) and 50% HAM’s F-10 with Glutamax-1 (Gibco, Cat. No 41-550, Life Technologies, Naerum, Denmark) with 10% Fetal Calf Serum (Gibco, Cat. No 10106-169, Life Technologies, Naerum, Denmark), 1% Penicillin G Sodium/Streptomycin Sulfate (Gibco, Cat. No 15140-122, Life Technologies, Naerum, Denmark), 300 μg/mL Geneticin Sulfate (Acros Organics, Cat. No 32940-0010, Life Technologies, Naerum, Denmark) and 1 ng/mL basic Fibroblast Growth Factor (Invitrogen, Cat. No 13-256-029, Naerum, Denmark). The medium was renewed every second day.

#### 2.4.2. Preparation of Plasmid

PHcRed1-C1 DNA (pDNA) encoding the red fluorescent protein HcRed was used as a reporter for evaluation of the transfection efficiency [8]. The pDNA was amplified in GC5TM competent *E. coli* cells (Sigma Aldrich, Cat. No G3169-10X50UL, Brondby, Denmark) and afterwards purified using a Nucleobond^®^ Xtra Midi kit (Macherey-Nagel, Cat. No 1101/003, Hojbjerg, Denmark) according to the manufacturer’s protocol.

#### 2.4.3. Magnetofection

The cells were seeded in a collagen-I coated 6-well plate (9.2 cm^2^/well) at a density of 300,000 cells/well (≈32,500 cells/cm^2^), incubated for 24 h at 37 °C, 5% CO_2_ in a humidified atmosphere in growth medium, and allowed to reach a confluence of 60%–70% before transfection assays was initiated. The medium was aspirated, and cells were gently washed twice in PBS. pDNA and chi-MNPs or lip-MNPs were then mixed in appropriate ratios (pDNA/MNP ratio of 1:50 and 1:46 *w*/*w*, respectively) in 200 µL serum and additive free medium consisting of 50% alpha-MEM and 50% HAM’s F-10. The solutions were allowed to incubate at ambient temperature for 20 min to ensure adequate complexation.

For the magnetofection procedure, the pre-incubated pDNA/MNP solutions were added to the wells in an amount of 2 µg pDNA per well irrespective of the vector tested. Serum and additive free medium were then added to reach a final volume of 2 mL, and the culture plates placed on a ferrite block magnet of 0.39 T for 20 min. Afterwards, the transfection medium was aspirated, the cells washed twice in PBS immediately following by addition of freshly prepared growth medium that was incubated with the cells for 48 h at 37 °C, 5% CO_2_ in a humidified atmosphere. An additional transfection assay was performed with the exception that magnetic field manipulation was left out during the transfection procedure. A commercially available reagent, TurboFect™ (Fermentas, Cat. No R0531, Waltham, MA, USA) was used as a positive transfection control. Transfections were assessed and evaluated by fluorescence microscopy (Carl Zeiss, Axiovert 200, Brock og Michelsen, Birkerod, Denmark). All experimental assays were performed in triplets.

#### 2.4.4. *In Vivo* Distribution

The distribution of chi-MNPs and lip-MNPs was studied in 15-day old Sprague Dawley rats (*n* = 4, mean body weight 35.75 ± 1.75 g), which received a single injection of 10 mg/kg of either compound in the tail vein. After 24 h, the rats were euthanized by an intraperitoneal injection of 0.3 mL Hypnorm/Dormicum (HYP/DOR) mixture and subsequently perfused with isotonic saline for 30 s followed by 4% paraformaldehyde (PFA) 0.1 M PBS, pH 7.4 for 10 min. The liver, spleen, kidneys, lungs, and brain were gently dissected and postfixed in 4% PFA at 4 °C overnight. Afterwards, the organs were dehydrated in a 30% sucrose solution for approximately 48 h, and the tissues cut into serial coronal 40 μm sections at −25 °C using a cryostat (Microm, HM 505N, Charleston, WV, USA). The sections were analyzed for the accumulation of non-heme iron of the magnetic particles using a protocol previously described [11]. In brief, the sections were cleansed twice times in KPBS followed by incubation in Perl’s solution (1:1 2% HCl and 2% Potassium hexacyanoferrate (II) trihydrate (Sigma Aldrich, Cat. No P9387-100G, Brondby, Denmark)) for 30 min at room temperature. Sections were then rinsed in distilled water and plated on glass slides for microscopic analysis. The procedures dealing with the handling of animals as described in this study were approved by the Danish National Council of Animal Welfare (permission no. 2006/561-1243).

For the distribution studies, a semi-quantification of non-heme iron accumulation as compared to the control rat was performed in a blinded manner on an arbitrary scale ranging from 0 (no difference), + (small increase), ++ (moderate increase) to +++ (large increase).

## 3. Results

### 3.1. Particle Characterization

**Figure 2:**
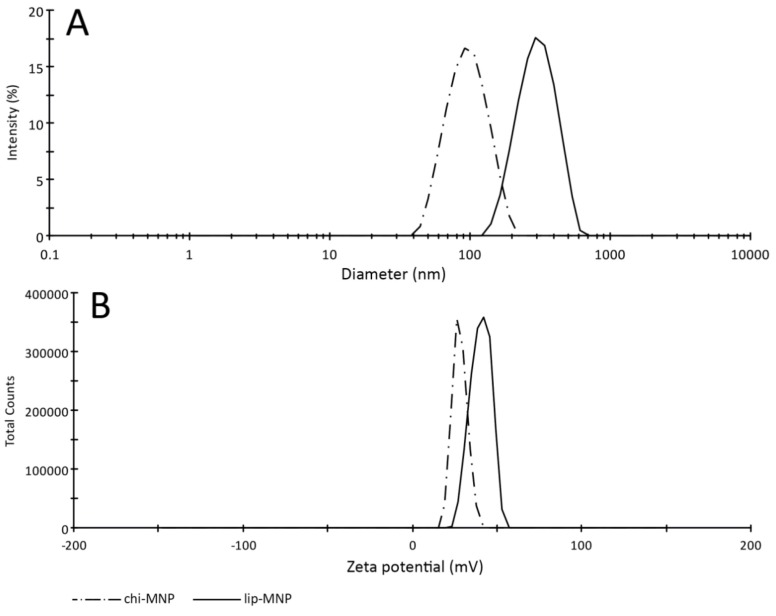
Size (**A**) and ζ-potential (**B**) characterization of nanoparticles. The size (nm) and ζ-potential (mV) was assessed using a Zetasizer Nano for chi-MNPs (dashed line) and lip-MNPs (solid line). The size (A) and ζ-potential (B) readings as assessed by the Zetasizer Nano. Triplet measurements were conducted for both size (nm) as well as ζ-potential for both chi-MNP (dashed line) and lip-MNP (solid line).

Analyses of the size and surface charge of the chi-MNPs showed that the mean hydrodynamic diameter of the particles was 103.62 ± 3.45 nm with a mean polydispersity index (PDI) of 0.195 ± 0.018. The mean ζ-potential of the chi-MNPs was 29.52 ± 1.79 mV (Figure 2A). Following synthesis of the lip-MNPs the mean hydrodynamic diameter and mean ζ-potential were found to be 303.37 ± 17.44 nm and 39.43 ± 1.59 mV, respectively (Figure 2B). Supplementary to the size determination of the lip-MNPs, the mean PDI was found to be 0.544 ± 0.19. Therefore, the lip-MNPs were determined to be approximately a 3-fold larger than the chi-MNPs, whereas the surface charge roughly was a 1.3-fold higher.

### 3.2. Assessment of DNA Binding Capacity

**Figure 3:**
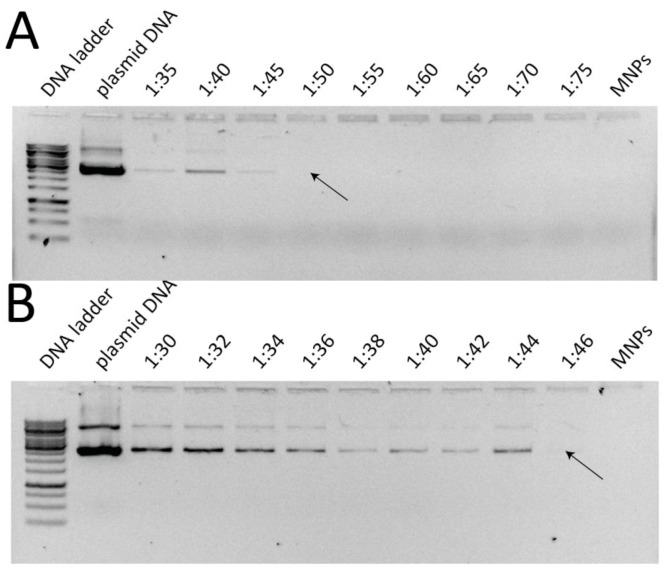
Assessment of DNA binding capacity of nanoparticles in gel-retardation assays. The assays were used to determine the pDNA/MBP ratio at which all pDNAs was bound to chi-MNPs (**A**) and lip-MNPs (**B**). Arrows indicate the ratio at where gel-bands representing pDNA were no longer visible indicative of a complete association between pDNA and the nanoparticle.

The DNA binding capacity of both the chi-MNPs and the lip-MNPs was evaluated in order to determine the highest pDNA/MNP ratio for each vector. Figure 3 shows the results of the gel retardation assay series. The highest pDNA/MNP (*w*/*w*) ratio for chi-MNPs was 1:50, whereas this ratio was 1:46 for the lip-MNPs, since the entire pDNA load was bound to the vectors at these ratios (Figure 3). These ratios were used for the magnetofection assays and indicated that the lip-MNPs were able to bind more DNA than chi-MNPs.

### 3.3. *In Vitro* Transfection Studies

**Figure 4:**
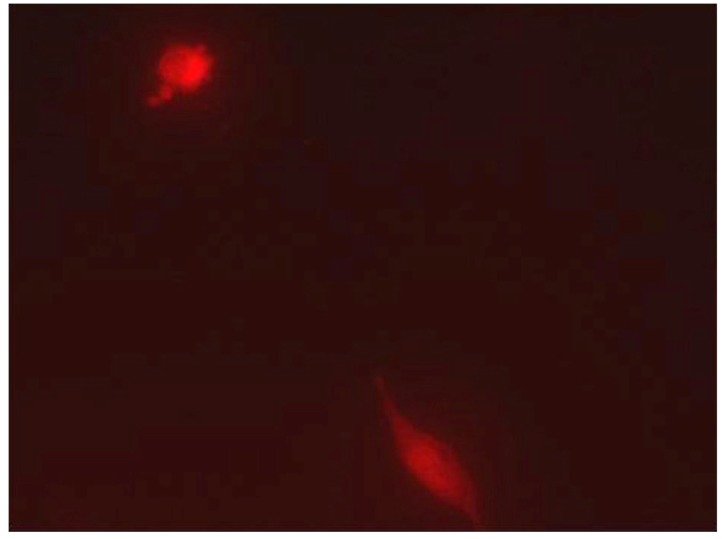
Transfection of RBE4 cells using pDNA-loaded lip-MNPs. The transfection efficiency of pDNA bound to MNPs is in the range of 20%–30% compared to that bound to Turbofect. The cells express the red fluorescent Hc-red protein in both the cell cytosol and nucleus.

Application of the magnetic force prominently increased the amount of particles present in the vicinity of the cultivated cells as compared to the situation where no magnetic field was applied clearly indicating the nanoparticles were mobile when subjected to the magnetic field. This observation was done for both chi-MNPs and lip-MNPs (not shown). Analysis of the magnetofection potential revealed a difference as a function of the magnetic vector. With the magnetic field applied, transfection was observed in cells treated with lip-MNPs (Figure 4), whereas no visible transfection was observed in wells containing cells added with chi-MNPs (data not shown). For both particle types, transfected cells could not be detected when the magnetic field manipulation was omitted (data not shown). Application of the commercial transfection kit TurboFect™ resulted in transfection within each well, which was estimated to be approximately five-fold higher than the transfection efficiency for the lip-MNPs.

### 3.4. Distribution Experiments

**Figure 5:**
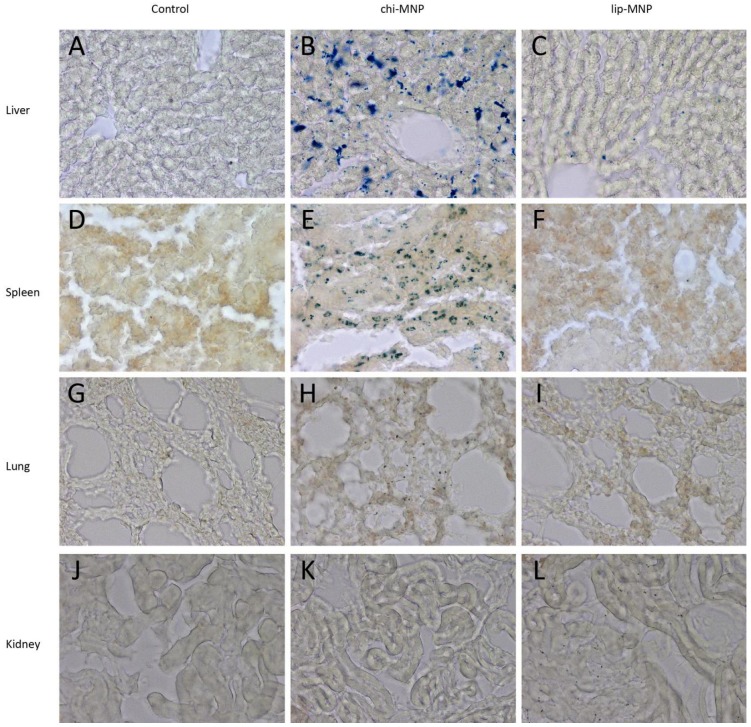
Representative images of columns are divided into three groups: non-injected controls, chi-MNP and lip-MNP. Rows are divided into organ-type: liver, spleen, lung and kidney. The control liver (**A**) was negative for non-heme iron whereas the chi-MNP liver was extensively positive for iron (**B**), which was displayed by an intense uneven blue colorization of the entire liver. Lip-MNP liver (**C**) showed only minute amounts of iron. The control spleen (**D**) as well as the lip-MNP spleen (**E**) was negative for the presence of iron whereas a strong positive reaction for iron was seen in the chi-MNP spleen (**F**). The control lung (**G**) was negative for non-heme iron. This was contrasted by the chi-MNP lung (**H**), which showed diffuse iron staining of the alveoli septa. Lip-MNP lung (**I**) did not show any reaction for iron. The control kidney (**J**) as well as the chi-MNP kidney (**K**) was negative for non-heme iron. Interestingly the lip-MNP kidney (**L**) showed a diffuse distribution of particulate shaped iron. Magnification: 400×.

**Figure 6:**
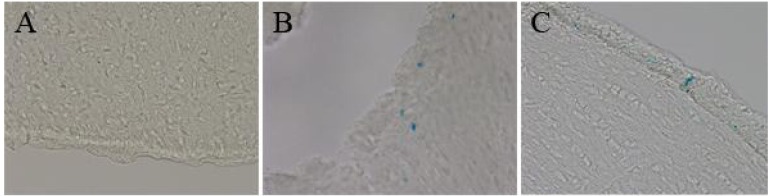
Iron-containing MNPs in brain. (**A**) Section from a non-injected brain. Without DAB-intensification, iron is not detected. On the contrary the brains of lip-MNP (**B**) and chi-MNP (**C**) injected animals displayed iron-containing MNPs. Magnification 400×.

To evaluate the distribution of chi-MNPs and lip-MNPs following intravenous injection, the brain, liver, spleen, kidneys, and lungs were dissected and analyzed. Due to the presence of autofluorescence within the sections mainly due to fixation with paraformaldehyde (not shown), the extent to which MNP accumulated in the various organs, sections were assessed by means of Perl’s staining for non-heme bound iron (Figure 5). Only negligible iron content could be detected in either of the organs examined in the control rats (Figure 5A,D,G,J). Compared to the control sections, the iron content in sections from rats injected with chi-MNP group was increased, especially in the liver and spleen (Figure 5B,E,H,K). The distribution of the lip-MNPs varied significantly from that of the chi-MNPs (Figure 5). Hence, only minute iron content could be detected in the liver, while iron virtually was absent in both the spleen and the lungs of the rats subjected to lip-MNPs indicated that the PEGylation of the particles lead to an escape from phagocytosis in the RES. In the brain, both chi-MNP and lip-MNPs were found in the walls of the ventricular system in contrast to that of the brain of non-injected rats, but chi-MNP was also detected in small amounts in the brain tissue distal to the ventricular system (Figure 6). Table 1 provides an overview of the relative iron content in each of the analyzed organs compared to the control group.

**Table 1:** Results of the graded comparison between the density of iron-labeling between the chi-MNP and lip-MNP group. The labeling was related to that of non-injected organs in which the endogenous iron-labeling was scored as the 0-value.

Organ	chi-MNP	Lip-MNP
Liver	+++	+
Spleen	+++	0
Lungs	+	0
Kidneys	0	+
Brain	+	+

The nomenclature refers to the following scenarios compared to that of non-injected organs: “0”: no difference, “+”: small increase, “++”: moderate increase, “+++”: large increase.

## 4. Discussion

The aim of the present study was to evaluate the magnetofection potential of two different MNP-based vectors; chi-MNPs and lip-MNPs. We demonstrate an enhanced magnetofection potential of lip-MNPs compared to unmodified chi-MNPs in an *in vitro* setting. A fundamental prerequisite for magnetofection is that the MNPs and the genetic material are able to sufficiently associate to form a stable complex, e.g., via non-specific electrostatic interactions. Thus, to ensure complexation with the anionic DNA we chose to use chi-MNPs, which due to its chitosan coating is cationic, hence enabling interaction with the DNA. For the same reason, we likewise aimed at developing lip-MNPs with an overall cationic surface charge.

Characterization of lip-MNPs revealed a 3-fold increase in the particle diameter and an approximately 1.3-fold increase in the ζ-potential, compared to the chi-MNPs, indicating that cationic lipids indeed encapsulated the lip-MNPs. The final net charge of the lip-MNPs seems strongly dependent on the lipid composition and both cationic and anionic lipids have previously been used for liposomal formulations. Pan *et al*. [10] showed that a lipid composition of DDAB and SOY PC in a molar ratio of 60:40 gave rise to particles with a ζ-potential of 85.54 ± 6.28 mV. In the present study, we used a somewhat similar lipid composition. However, we experienced an approximate 2-fold decline in the surface charge of lip-MNPs compared to the findings of Pan *et al*. [10]. This could be explained by the incorporation of the anionic lipid DSPE-PEG2000-MAL in the liposome bilayer, which lowers the surface charge of the liposome, emphasizing the importance of the lipid composition. Hartig *et al*. [12] proposed that an optimal particle should have a surface charge of >30 mV in order to provide the most beneficial cellular uptake, likely because the cationic charge promotes increased interaction with the negatively charged cell membranes. Based on this, both of the vectors used in the present study seem optimal with regards to surface charge.

Apart from the above, several other features like the size and the DNA binding capacity are vital to consider in the process of designing a new vector for gene delivery. Senyei *et al*. [13] previously stipulated several important properties, which if fulfilled, would give rise to an optimal MNP for drug delivery purposes. Especially the suggestions regarding particle diameter seem of utmost importance to ensure both efficacy and safety. The importance of the particle size for efficacy is twofold: Utilization of small particles allows capillary-level distribution, which in turn ensures uniform perfusion of the target tissue. Furthermore, a relatively small particle diameter is warranted for *in vivo* applications because this diminishes the risk of emboli formation within the cardiovascular system. Concerning the latter, it was proposed that the critical cut-off size is 5 µm, hence well-above the siz of the MNPs used in the present study, which was approximately 100 nm and 300 nm for chi-MNPs and lip-MNPs, respectively. Nevertheless, no simple rule seems applicable in dictating the optimal particle size, since this varies with the core material and the application purposes of the MNP.

### 4.1. *In Vitro* Magnetofection

In the present study we demonstrated that transfection of RBE4 cells mediated by lip-MNPs resulted in expression of the far-red fluorescent protein HcRed. On the contrary, chi-MNPs were unsuccessful in transfecting RBE4 cells. These results are supported by the findings of other authors, who demonstrated a significant transfection potential of lipid-encapsulated MNPs and further in line with our results, Kievit *et al*. showed that chi-MNPs failed to mediate transfection of cells *in vitro* [9].

The influence of a magnetic field on the transfection efficiency has been investigated by numerous studies and it has been reported to increase the transfection efficiency by a 100- to 1000-fold. This increase in the transfection efficacy may be explained by alterations in the sedimentation kinetics of the particles under these circumstances and indeed several studies acknowledge sedimentation and internalization as critical steps for successful transfection. The sedimentation step is typically diffusion-limited, however, during magnetofection MNP-to-cell contact is greatly enhanced by magnetic sedimentation, which annuls this diffusion limit, promotes MNP-to-cell contact, and thereby enhances the subsequent internalization of the pDNA-MNP complex. Interestingly, when we investigated the internalization of lip-MNPs versus chi-MNPs, in the presence or absence of a magnetic field, it was observed that both the lipid-encapsulation and magnetic exposure seemed to serve crucial roles in predicting the amount of pDNA-MNP complex internalized by the cells. Several mechanisms may act symbiotically to facilitate this increased internalization of lip-MNPs. A study conducted by Prow *et al*. [14] demonstrated that increases in size and mass of a nanoparticle improve the likelihood of particle-to-cell contact, a prerequisite for internalization. Therefore, the larger size and higher mass of the lip-MNPs may increase their sedimentation rate and subsequent internalization, compared to chi-MNPs. Another supplementary explanation may be found in lipid-specific endocytotic mechanisms. The endocytosis of cationic particles depends largely on adsorptive mediated endocytosis. Nonetheless, the lip-MNPs may be superior compared to chi-MNPs in exploiting such a transport mechanism, because of their lipid-encapsulation and increased surface charge, which might favor association with the anionic cell membrane. In addition, it could be speculated that the lipid bilayer provides an ability to fuse with the cell membrane increasing the efficiency by which the particle is endocytosed. Noteworthy, the MNPs do not lead to detectable toxicity of the cells [5].

PEGylated liposomes suffer from the drawback of being less efficient as transfection agents, which are likely due to that PEG may impair electrostatic interactions between the cationic lipids and the DNA [15]. PEG provides shielding of the particle from the surroundings and thereby also restricts uptake in the cultivated cells PEG may impair pDNA-MNP dissociation after endocytosis and thereby hinder the pDNA in entering the nucleus. Other authors have demonstrated a pDNA:chi-MNP ratio of 1:10 (*w*/*w*) for PEGylated complexes , whereas there to our knowledge is no comparable data for the lip-MNPs. As presented by Mykhaylyk *et al*. [16], it is of utmost importance to determine the pDNA:MNP ratio at which the highest transfection is achieved.

### 4.2. *In Vivo* Distribution of Magnetic Nanoparticles

The results obtained from the distribution study demonstrated that the two magnetic vectors indeed had very different pharmacokinetic profiles, reflected by their distinct distributional patterns. The chi-MNPs could predominantly be detected in the liver and spleen with a smaller fraction observed in the lungs and the brain. The body distribution of lip-MNPs was found to be largely different from these findings, since only minute particle amounts could be detected in the liver and brain, whereas the spleen and the lungs remained principally free of particle deposits. Only lip-MNPs were observed in the kidneys.

The very distinct distribution patterns associated with these two different delivery systems is not trivial and accordingly several studies report that altered body distribution is likely to occur as a function of the size, charge, and surface coating of the injected nanoparticles. The distribution of PEGylated liposomes significantly differed from that of non-PEGylated liposomes [17]. The authors performed an isolated liver perfusion technique and found that the non-PEGylated liposomes showed substantial sinusoidal uptake, whereas the PEGylated liposomes predominantly remained in the circulatory system. It is furthermore widely accepted that following intravenous injection, non-PEGylated nanoparticles tend to accumulate in the spleen, due to extensive particle uptake by the residing macrophages. Both of these observations are in concordance with the results obtained in the present study. Moreover, since the lip-MNPs were PEGylated, which is known to prolong the *in vivo* blood circulation time, this might provide an explanation for the uniquely observed lip-MNP deposits in the kidney sections. This observation has previously been reported for liposome formulations with prolonged circulation time.

The RES exhibits an inherent ability to recognize and sequester nanoparticles from the circulation, which severely impedes the nanoparticle in reaching its target destination. This unspecific RES uptake is seen virtually within minutes after intravenous injection and has been determined to be a consequence of adsorption of plasma proteins and other blood components to the nanoparticle, which renders an increased recognition and uptake in macrophages likely. The extent of plasma protein adsorption to nanoparticles has been shown to correlate with the size of the particles [7]. Based on this, larger particles are more prone to RES sequestering from the blood stream and indeed large particles have been shown to suffer from a rapid plasma clearance. These findings are rather inconsistent with the findings from the present study, since the smaller chi-MNPs (≈100 nm) seemed more prone to RES uptake, as represented by the increased deposits in the liver and spleen, compared to the larger lip-MNPs (≈300 nm). The explanation for this phenomenon is likely to be found in the protective effects exhibited by the PEGylation of the lip-MNPs. PEGylation of liposome formulations ensures sterically stable liposomes, which has been described as “stealth liposomes”, due to their ability to avoid the RES, a trait attributed to steric hindrance of plasma protein adsorption to the nanoparticle surface. Finally, we propose that another mechanism may exist, which further assists in prolonging the circulation time of the lip-MNPs. As explained earlier, the lipid-encapsulation of the MNPs in the present study resulted in a prominent diameter increase of the particles. The increased diameter of the particles is likely to prevent extravasation of these particles in the spleen and the liver, since the fenestrae of the vascular system in these tissues have been determined to be <150 nm. Thus, both the size and the surface coating, and especially the presence of PEG, of the lip-MNPs are likely to provide an explanation for the low RES uptake, as reported in this study.

### 4.3. Targeted Therapy Using Magnetic Nanoparticles

At present, the major obstacle in the treatment of a large variety of CNS disorder is the blood-brain barrier, which severely hampers adequate delivery of therapeutic substances to the CNS parenchyma [18]. The use of colloid-based drug and gene delivery systems is an emerging area, which offers the possibility to significantly enhance the impaired delivery and thereby refine the treatment options for CNS diseases. Magnetic nanoparticles are an example of one such colloid-based carrier that, due to their unique structure, exhibit an inert capacity to associate with a plethora of drugs and genetic material and additionally offer targeted delivery through their inherent magnetic properties. The above emphasizes that by specific surface coat tailoring, manipulation of the biodistribution can be achieved and the effective circulation time can be prolonged. This is an important feature for e.g., antibody-mediated targeted delivery, since it offers a prolonged time window, where the antibody can interact with its target molecule. When the objective is to target the blood-brain barrier, as in this study, conjugation of an antibody specific for a molecule exclusively expressed on the brain capillary endothelial cells is the natural choice. One such target is the transferrin receptor, and indeed the anti-transferrin receptor antibody OX-26 has previously been used for this purpose. Conjugation of the OX-26 molecule to the lip-MNPs synthesized in this study should be possible, since the lip-MNPs were designed with this idea in mind and therefore DSPE-PEG2000-MAL was incorporated in the lipid composition. Gosk *et al*. have previously shown that the OX-26 antibody can be conjugated to this component of a liposome [19], which may pave the way for increasing the MNP dose delivered to the central nervous system in future experiments using targeted therapy.

## 5. Conclusions

The overall purpose of the experiments performed in this study was to evaluate the transfection potential of the commercial available chi-MNPs, compared to self-synthesized liposome-encapsulated MNPs. In conclusion, the results showed that we were able to synthesize a novel carrier that exhibited the characteristics required for carrying and delivering genetic material. Furthermore, it was evident that the lip-MNPs are superior with regards to transfection of brain endothelial cells *in vitro*, compared to the chi-MNPs. The *in vivo* studies revealed that lip-MNPs had a superior pharmacokinetic profile, compared to chi-MNPs, likely due to evasion of the RES.

## Abbreviations

chi-MNPs: chitosan-coated magnetic nanoparticle
DDAB: dimethyldioctadecylammonium bromide
DSPE-PEG2000-MAL: 1,2-distearoyl-*sn*-glycero-3-phosphoethanolamine-*N*-[maleimide (polyethylene glycol)-2000]
DLS: dynamic light scattering
MNP: magnetic nanoparticle
pDNA: plasmid pHcRed1-C1
RBE4: rat brain endothelial 4
RES: reticuloendothelial system
Soy PC: l-α-phosphatidylcholine
